# Supplementary material for: Colonic inflammation modulates the intestinal circadian landscape
Source: iScience. 2025 Jul 23;28(8):113183. doi: 10.1016/j.isci.2025.113183 (PMC12355569; doi:10.1016/j.isci.2025.113183)
Supplement: Document S1. Figures S1–S8 and Tables S1–S3 and S5 [file mmc1.pdf]

iScience, Volume *28*

Supplemental information

Colonic inflammation modulates

the intestinal circadian landscape

Thomas D. Butler, Polly Downton, Suzanna H. Dickson, Andrea Luengas-Martinez, Devin A. Simpkins, Isabel Khoo, Sarah Veal, Alexander C. West, Antony D. Adamson, David A. Bechtold, John T. McLaughlin, and Julie E. Gibbs

Table S1. Genes included in pathway analysis

KEGG 2019 mouse Pathway	Number of genes	Genes in pathway
IBD (mmu05321)	54	foxp3, h2-aa, h2-ab1, h2-dma, h2-dmb1, h2-dmb2, h2-eb1, h2-oa, h2-ob, ifng, ifngr1, ifngr2, il10, il12a, il12b, il12rb1, il12rb2, il13, il17a, il17f, il18, il18r1, il18rap, il1a, il1b, il21, il21r, il22, il23a, il23r, il2rg, il4ra, jun, maf, nfatc1, nfkb1, nod2, rela, rora, rorc, smad2, smad3, stat1, stat3, stat4, stat6, tbx21, tgfb1, tgfb2, tgfb3, tlr2, tlr4, tlr5, tnf
Intestinal immune network for IgA production (mmu04672)	37	aicda, ccl25, ccl28, ccr10, ccr9, cd28, cd40, cd40lg, cd80, cd86, cxcl12, cxcr4, h2-aa, h2-ab1, h2-dma, h2-dmb1, h2-dmb2, h2-eb1, h2-oa, h2-ob, icos, icosl, il10, il15, il15ra, itga4, itgb7, ltbr, madcam1, map3k14, pigr, tgfb1, tnfrsf13b, tnfrsf13c, tnfrsf17, tnfsf13, tnfsf13b
Antigen processing and presentation (mmu04612)	71	b2m, calr, canx, cd4, cd74, cd8a, cd8b1, ciita, creb1, ctsb, ctsl, ctss, gm7030, gm8909, h2-aa, h2-ab1, h2-bl, h2-d1, h2-dma, h2-dmb1, h2-dmb2, h2-eb1, h2-k1, h2-m2, h2-m3, h2-m5, h2-m9, h2-oa, h2-ob, h2-q1, h2-q10, h2-q2, h2-q4, h2-q6, h2-q7, h2-t-ps, h2-t10, h2-t22, h2-t23, h2-t24, h2-t3, hsp90aa1, hsp90ab1, hspa1a, hspa1b, hspa1l, hspa2, hspa4, hspa5, hspa8, ifi30, ifng, klrc1, klrc2, klrd1, lgmn, nfya, nfyb, nfyc, pdia3, psme1, psme2, psme2b, psme3, rfx5, rfxank, rfxap, tap1, tap2, tapbp, tnf
Tight junction (mmu04530)	147	actb, actg1, actn1, actn4, actr2, actr3, actr3b, afdn, amot, amotl1, amotl2, arhgap17, arhgef18, arhgef2, bves, cacna1d, ccnd1, cd1d1, cdc42, cdk4, cftr, cgn, cgnl1, cldn1, cldn10, cldn11, cldn14, cldn15, cldn2, cldn20, cldn22, cldn23, cldn3, cldn4, cldn5, cldn7, cldn8, cldn9, crb3, cttn, dlg1, dlg2, dlg3, epb41l4b, erbb2, ezr, f11r, gata4, hcls1, hspa4, igsf5, itgb1, jam2, jam3, jun, llgl1, llgl2, magi1, map2k7, map3k1, map3k5, mapk10, mapk8, mapk9, marveld2, marveld3, micall2, mpdz, mpp4, msn, myh10, myh11, myh14, myh2, myh3, myh7b, myh9, myl12a, myl12b, myl6, myl6b, myl9, nedd4, nedd4l, nf2, ocln, pard3, pard6a, pard6b, pard6g, patj, pcna, ppp2ca, ppp2cb, ppp2r1a, ppp2r1b, ppp2r2a, ppp2r2b, ppp2r2c, ppp2r2d, prkaa1, prkaa2, prkab1, prkab2, prkaca, prkacb, prkag1, prkag2, prkag3, prkce, prkci, prkcz, rab13, rab8a, rab8b, rac1, rap1a, rap2c, rapgef2, rapgef6, rdx, rhoa, rock1, rock2, runx1, scrib, slc9a3r1, src, stk11, sympk, synpo, tiam1, tjap1, tjp1, tjp2, tjp3, tuba1a, tuba1b, tuba1c, tuba4a, tuba8, tubal3, vasp, was, wasl, whamm, ybx3

Table S2: Baseline cytokine levels from multiplex immunoassay

	Bmal1^{flox}		IEC-Bmal1$^{-/-}$		
**Cytokine**	**Mean** (pg/ml)	**SEM** (pg/ml)	**Mean** (pg/ml)	**SEM** (pg/ml)	**p value**
**Eotaxin**	3444.8	309.9	3529.4	650.8	0.90
**G-CSF**	151.5	45.0	163.1	50.2	0.87
**GM-CSF**	58.0	10.5	50.0	15.0	0.67
**IFN-γ**	62.3	32.4	34.3	7.3	0.48
**IL-1α**	153.2	35.0	65.3	6.2	0.06
**IL-1β**	19.0	2.6	9.3	3.0	0.06
**IL-2**	13.1	1.7	12.5	1.3	0.79
**IL-3**	11.1	1.1	9.6	2.8	0.60
**IL-4**	15.5	1.5	12.7	2.7	0.38
**IL-5**	18.5	3.4	17.1	5.3	0.83
**IL-6**	8.4	4.6	11.7	4.7	0.64
**IL-9**	49.3	23.2	27.6	5.6	0.44
**IL-10**	65.1	5.4	246.5	181.1	0.29
**IL-12(p40)**	1122.2	108.4	1033.0	96.0	0.57
**IL-12(p70)**	208.7	32.6	219.3	52.0	0.86
**IL-13**	77.7	15.0	52.5	9.1	0.22
**IL-17A**	194.6	34.0	170.1	35.2	0.63
**CXCL1 (KC)**	143.4	51.6	172.0	27.0	0.66
**MCP-1**	477.4	110.1	583.0	131.4	0.55
**MIP-1α**	8.4	0.8	5.9	0.8	0.06
**MIP-1β**	61.2	4.7	46.8	3.8	0.06
**CCL5 (RANTES)**	291.2	24.2	216.7	23.3	0.07
**TNF-α**	153.7	15.4	181.3	58.1	0.62

Table S3: DSS colitis scoring system

Score	Weight loss	Stool consistency	Bleeding
0	0	Normal	No blood
1	5-10%	-	-
2	10-15%	Loose	Visible blood on pellet
3	15-20%	-	-
4	>20%	Diarrhoea	Gross bleeding with blood around anus

Table S4. Summary of statistical tests and results

Table S4 is presented in a separate .xls file

Table S5: *qPCR* primers and probes

Gene	Forward primer	Reverse primer	Fam-Tamra probe
Gapdh	CAA TGT GTC CGT CGT CGA TCT	GTC CTC AGT GTA GCC CAA GAT G	CGT GCC GCC TGG AGA AAC CTG CC
Rps18	Proprietary assay (Mm02601777_g1)		
β-actin	AGG TCA TCA CTA TTG GCA ACG A	CAC TTC ATG ATG GAA TTG AAT GTA GTT	TGC CAC AGG ATT CCA TAC CCA AGA AGG
Cre	Proprietary assay (Mr00635245_cn)		
Bmal1 (exon 8)	CGT CGG GAC AAA ATG AAC AG	GAA CAG CCA TCC TTA GCA C	TAC CCA CAT GCA ATG CAA TGT CCA GGA A
Nr1d1 (Reverbα)	Proprietary assay (Mm00520708_m1)		
Per2	GCC TTC AGA CTC ATG ATG ACA GA	TTT GTG TGC GTC AGC TTT GG	ACT GCT CAC TAC TGC AGC CGC TCG T
Cry1	CTGGCGTGG AAGTCATCGT	CTGTCCGCCATT GAGTTCTATG	CGCATTTCACATACACT GTATGACCTGGACA
Il1β	TCG CTC AGG GTC ACA AGA AA	CCA TCA GAG GCA AGG AGG AA	CAT GGC ACA TTC TGT TCA AAG AGA GCC TG
Il6	CTA TAC CAC TTC ACA AGT CGG AGG	TGC ACA ACT CTT TTC TCA TTT CC	TTA ATT ACA CAT GTT CTC TGG GAA ATC G
Il10	Proprietary assay (Mm01288386_m1)		
Tnfα	TCT CTT CAA GGG ACA AGG CTG	ATA GCA AAT CGG CTG ACG GT	CCC GAC TAC GTG CTC CTC ACC CA
Ifnγ	TCA AGT GGC ATA GAT GTG GAA GAA	TGG CTC TGC AGG ATT TTC ATG	TCA CCA TCC TTT TGC CAG TTC CTC CAG
Cxcl1	CTG CAC CCA AAC CGA AGT	AGC TTC AGG GTC AAG GCA AG	CAC TCA AGA ATG GTC GCG AGG C
Cxcl5	Proprietary assay (Mm00436451_ g1)		
Ccl2	TTC TGG GCC TGC TGT TCA	CCA GCC TAC TCA TTG GGA TCA	CTC AGC CAG ATG CAG TTA ACG CCC C

Figure S1

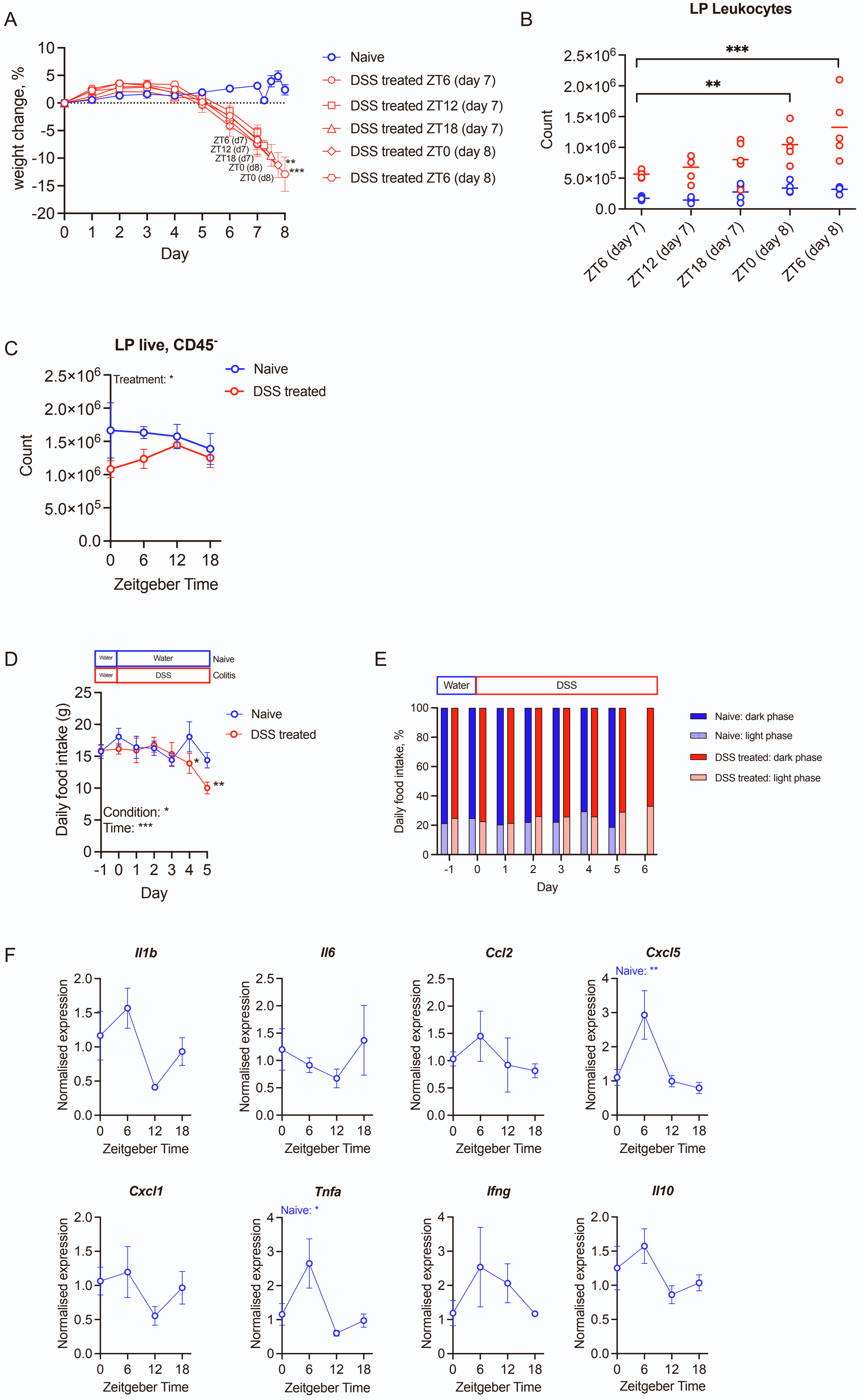
A
weight change, %
Day
ZT6 (d7)
ZT12 (d7)
ZT18 (d7)
ZT0 (d8)
ZT0 (d8)
Naive
DSS treated ZT6 (day 7)
DSS treated ZT12 (day 7)
DSS treated ZT18 (day 7)
DSS treated ZT0 (day 8)
DSS treated ZT6 (day 8)
B
LP Leukocytes
Count
ZT6 (day 7)
ZT12 (day 7)
ZT18 (day 7)
ZT0 (day 8)
ZT6 (day 8)
C
LP live, CD45-
Treatment: *
Naive
DSS treated
Count
Zeitgeber Time
D
Water
Water
Naive
Water
DSS
Colitis
Daily food intake (g)
Condition: *
Time: ***
Day
E
Water
DSS
Daily food intake, %
Day
Naive: dark phase
Naive: light phase
DSS treated: dark phase
DSS treated: light phase
F
Il1b
Il6
Ccl2
Cxcl5
Naive: **
Cxcl1
Tnfa
Naive: *
Ifng
Il10
Normalised expression
Zeitgeber Time

Figure S1: Acute DSS induced colitis exhibits circadian characteristics. (A) Percentage weight change of mice exposed to water or 2.5% DSS initiated at the same time, Zeitgeber Time (ZT)4, where ZT0 represents lights on, and ZT12 represents lights off. Data were normalised to day 0 weight (DSS treated, n=5/timepoint; Naïve controls, n=5/timepoint (data pooled into one group). **(B)** Number of lamina propria (LP) leukocytes (live $CD45^+$), assessed by flow cytometry, n=5/timepoint/treatment. **(C)** Flow cytometry data showing number of live $CD45^-$ cells in the lamina propria across time in naïve and DSS treated (colitis) mice, n=5/treatment/timepoint. **(D)** Daily food intake prior to the initiation of colitis and during DSS treatment (per cage, n=4/cage, 1 cage/treatment) **(E)** Day/night food intake (per cage, n=4/cage, 1 cage/treatment) as a percentage of total daily food intake. **(F)** qPCR data from naïve colon samples collected at day 7, represented as fold change in ΔΔCt values, using naïve ZT0 as referent population and *B-actin* as housekeeping gene. N=4-5/timepoint. Statistics: (A) Two-way ANOVA with multiple comparisons (Šídák). * = compared with colitis ZT6 (day 7). (B) Two-way ANOVA with multiple comparisons (Dunnett) (C) Two-way ANOVA with multiple comparisons (Šídák). (C and F) Nonlinear regression to compare whether best fit is given by horizontal line or sine wave with nonzero baseline, constraints: wavelength = 24 hours; amplitude > 0. P value for sine wave displayed in coloured text on plots, where significant. (D) Two-way ANOVA with multiple comparisons (Tukey).

Figure S2

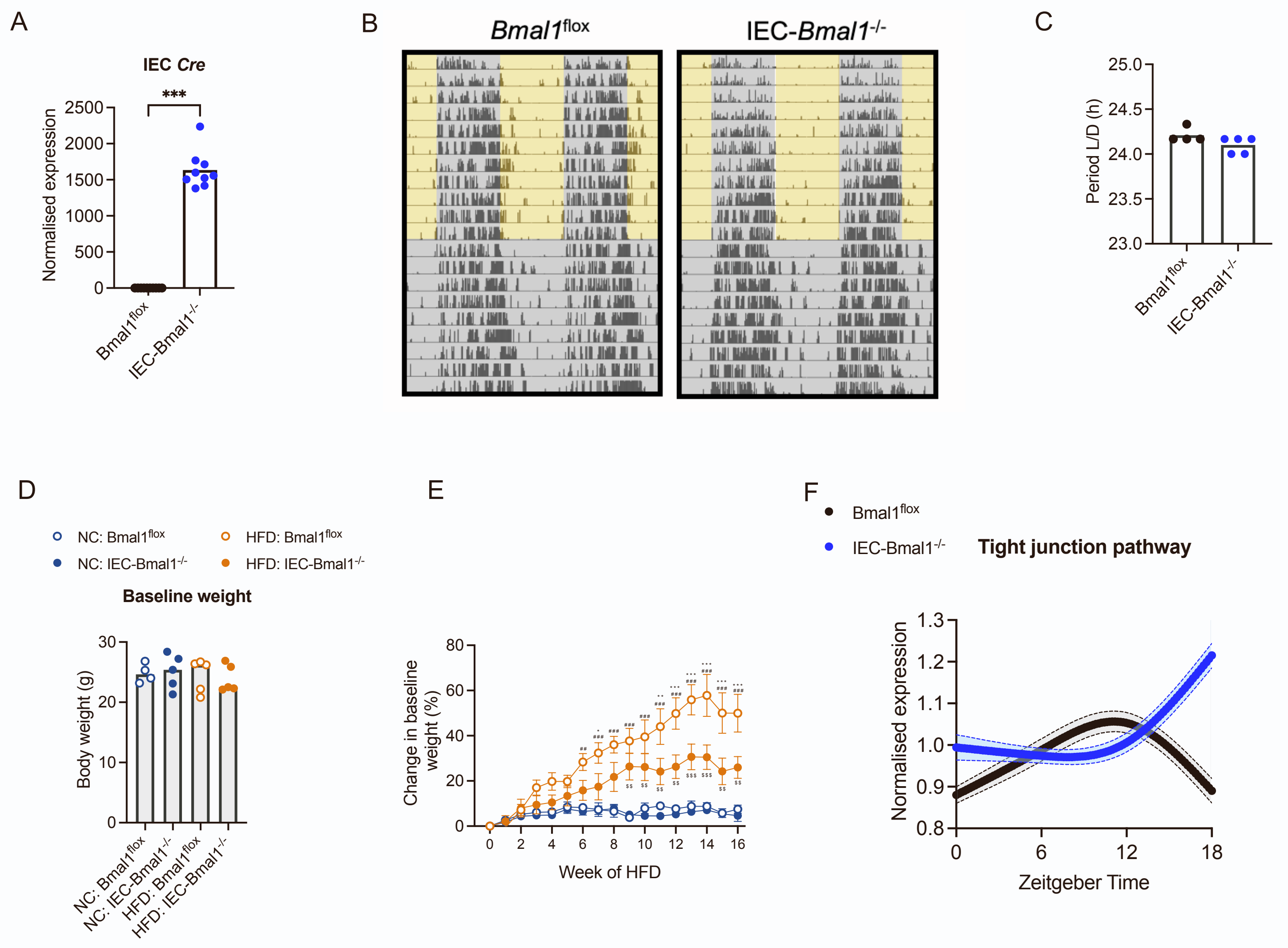
A
IEC Cre
***
Normalised expression
2500
2000
1500
1000
500
0
Bmal1flox
IEC-Bmal1-/-
B
Bmal1flox
IEC-Bmal1-/-
C
Period L/D (h)
25.0
24.5
24.0
23.5
23.0
Bmal1flox
IEC-Bmal1-/-
D
NC: Bmal1flox
NC: IEC-Bmal1-/-
HFD: Bmal1flox
HFD: IEC-Bmal1-/-
Baseline weight
Body weight (g)
30
20
10
0
E
Change in baseline weight (%)
80
60
40
20
0
Week of HFD
F
Bmal1flox
IEC-Bmal1-/-
Tight junction pathway
Normalised expression
1.3
1.2
1.1
1.0
0.9
0.8
0
6
12
18
Zeitgeber Time

Figure S2: IEC-specific *Bmal1* deletion alters the colonic transcriptome. (A) Colonic intestinal epithelial cell (IEC) *Cre* gene expression in naïve mice, represented as fold change in ΔΔCt values, using naïve ZT0 group as referent population and *Gapdh* as housekeeping gene, n=9-10/genotype. **(B)** Representative wheel-running actogram in light/dark (Day 1-11) and constant dark (Day 12-20) conditions. **(C)** Actogram-derived period length during light/dark conditions, n=4-5/genotype. **(D)** Baseline body weight of animals prior to high fat diet (HFD; n=4-5/genotype) or for controls normal chow (NC; n=5/genotype). **(E)** Percentage weight change in mice during 16 week HFD or NC, normalised to baseline weight. * Significance vs. HFD: IEC-*Bmal1*$^{-/-}$; $^{\#}$ Significance vs. NC: *Bmal1*^{flox}; $^{\$}$ Significance vs. NC: IEC-*Bmal1*$^{-/-}$. **(F)** Spline plots showing mean normalised expression of all genes from the dataset within the tight junction pathway (mmu04530 147/169), error bars represent 95% confidence intervals. Statistics: (A) unpaired two-tailed t test (C) unpaired two-tailed t test (D) One-way ANOVA (E) Two-way ANOVA with multiple comparisons (Tukey).

Figure S3

A

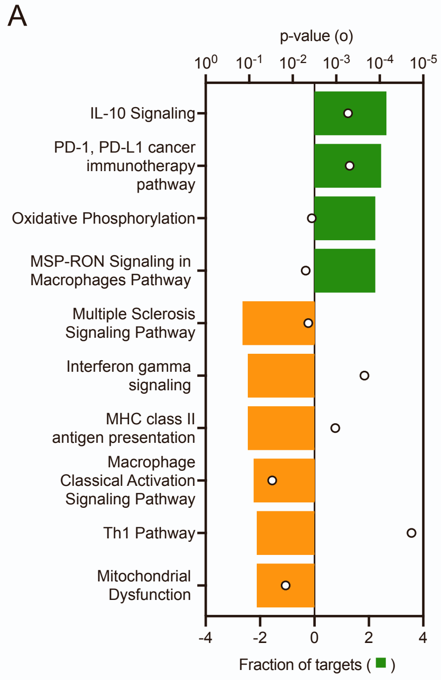

B

Diseases or Functions Annotation	p-value
Chronic colitis	1.82E-12
Experimental colitis	3.80E-12
Epithelial neoplasm	3.99E-11
Non-melanoma solid tumor	7.61E-11
Carcinoma	1.19E-10
Endocrine gland tumor	1.23E-10
Non-hematological solid tumor	1.89E-10
Endocrine carcinoma	3.10E-10
Dextran sodium sulfate-induced colitis	3.94E-10
Nonpituitary endocrine tumour	4.78E-10

C

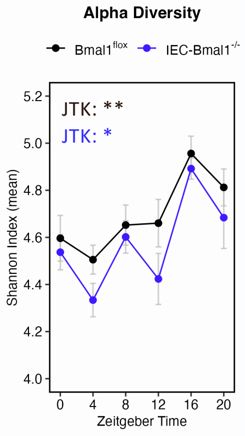

D

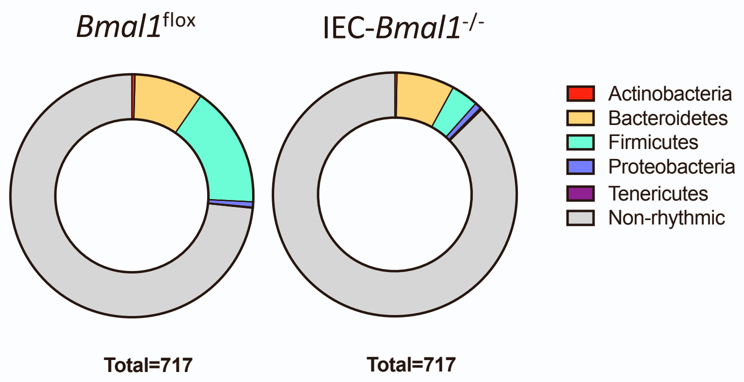

E

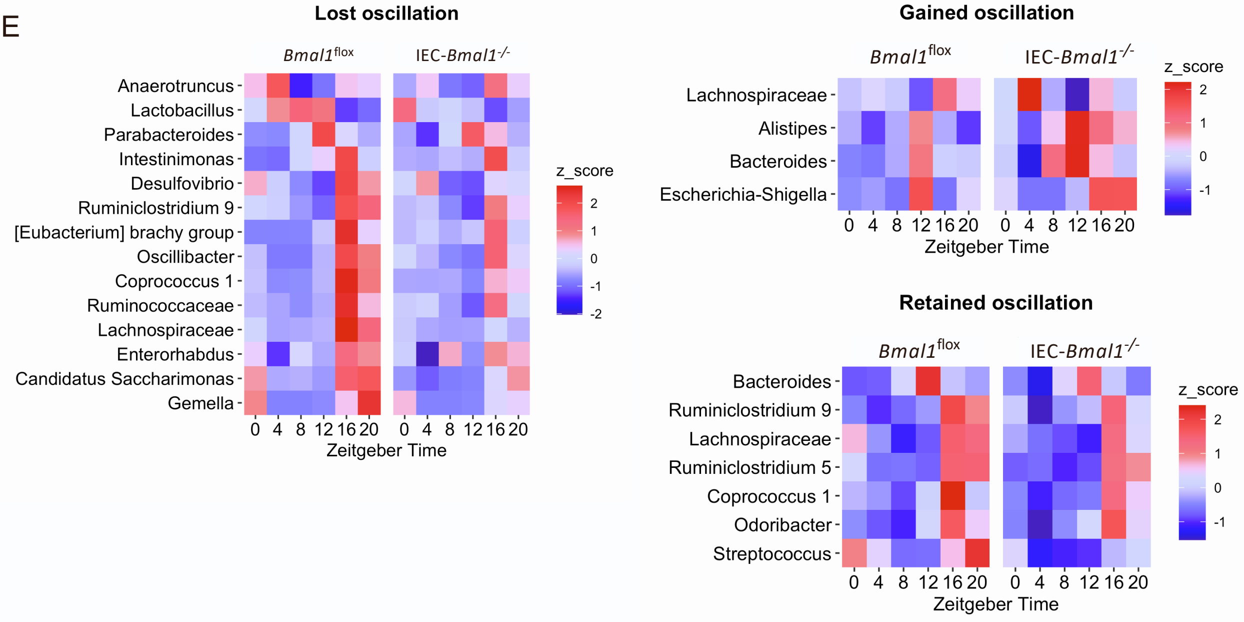

Figure S3: IEC-specific *Bmal1* deletion alters the colonic rhythmic transcriptome, rhythmic microbiome and key immune pathways. (A) Ingenuity pathway analysis of significantly activated (green, z score >2) and inhibited (orange, z score > -2) pathways in colonic transcriptome from IEC-*Bmal1*$^{-/-}$ mice compared to *Bmal1*^{flox}. **(B)** A table of the top 10 disease annotations significantly impacted in IEC-*Bmal1*$^{-/-}$ mice compared to *Bmal1*^{flox}. **(C)** Alpha diversity within 16S microbiome sequencing (n=30/genotype). **(D)** Rhythmic operational taxonomic unit (OTUs) categorised by phyla. **(E)** Heatmaps of comparative rhythmicity for annotated genera. Statistics: (C and E) Analysis of rhythmicity by JTK_CYCLE.

Figure S4

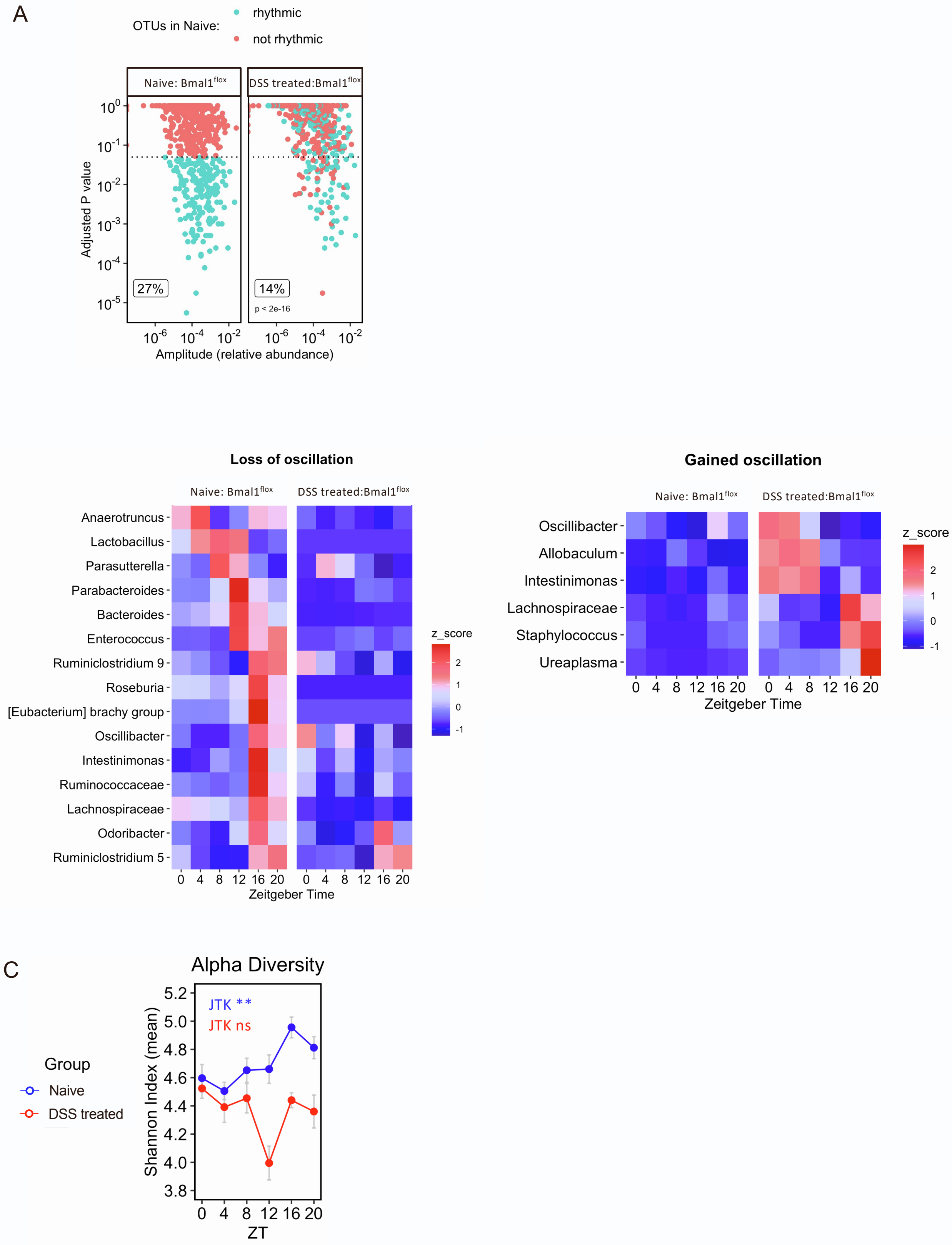
A
OTUs in Naive:
rhythmic
not rhythmic
Naive: Bmal1flox
DSS treated:Bmal1flox
Adjusted P value
27%
14%
p < 2e-16
Amplitude (relative abundance)
Loss of oscillation
Naive: Bmal1flox
DSS treated:Bmal1flox
Anaerotruncus
Lactobacillus
Parasutterella
Parabacteroides
Bacteroides
Enterococcus
Ruminiclostridium 9
Roseburia
[Eubacterium] brachy group
Oscillibacter
Intestinimonas
Ruminococcaceae
Lachnospiraceae
Odoribacter
Ruminiclostridium 5
z_score
Zeitgeber Time
Gained oscillation
Naive: Bmal1flox
DSS treated:Bmal1flox
Oscillibacter
Allobaculum
Intestinimonas
Lachnospiraceae
Staphylococcus
Ureaplasma
z_score
Zeitgeber Time
C
Alpha Diversity
Group
Naive
DSS treated
JTK **
JTK ns
Shannon Index (mean)
ZT

Figure S4: DSS treatment alters the colonic rhythmic microbiome. (A) Rhythmicity in relative abundance of operational taxonomic units (OTUs) in faecal samples harvested around the clock from *Bmal1*^{flox} mice prior to and during (Day 4) DSS colitis (paired samples), n=5/timepoint. **(B)** Heatmaps of comparative rhythmicity for annotated genera. **(C)** Alpha diversity within 16S microbiome sequencing (n=5/treatment/timepoint). Statistics: (A-C) Analysis of rhythmicity by JTK_CYCLE. (A) McNemar test.

Figure S5

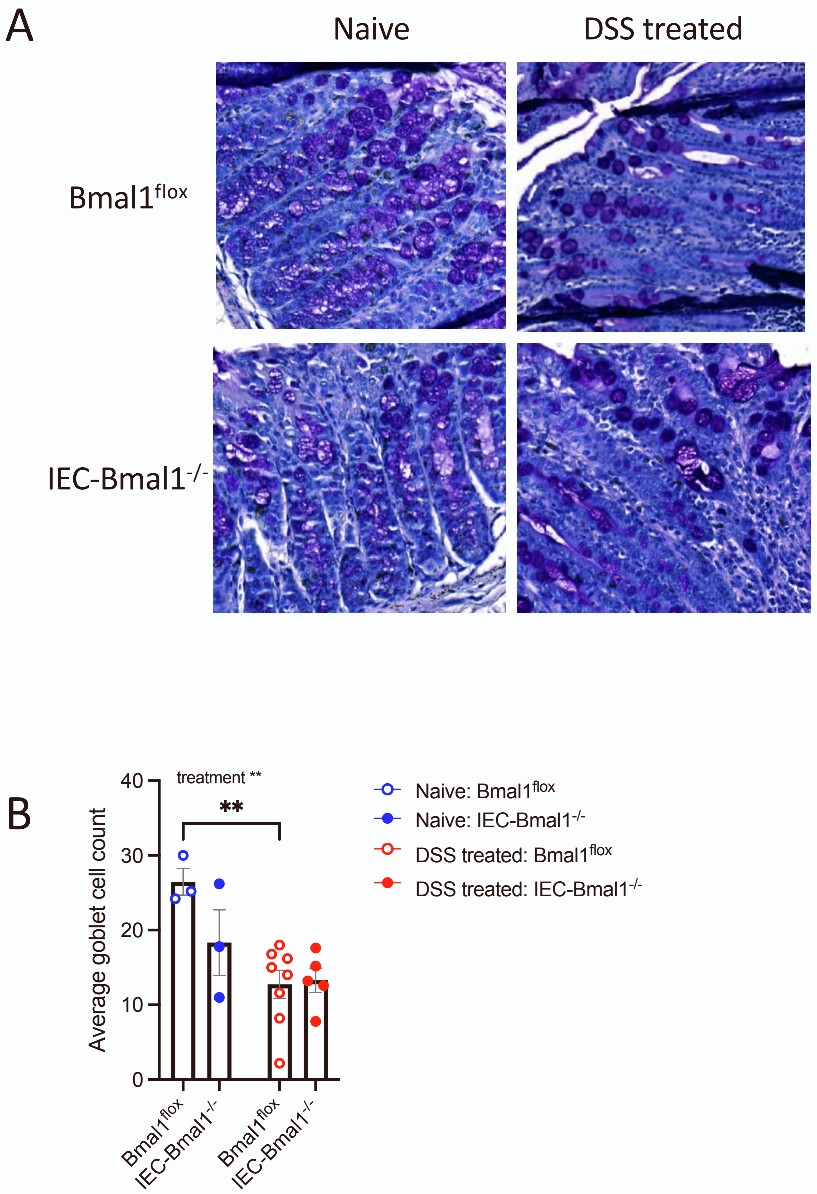
A
Naive
DSS treated
Bmal1flox
IEC-Bmal1-/-
B
Average goblet cell count
treatment **
**
0
10
20
30
40
Bmal1flox
IEC-Bmal1-/-
Bmal1flox
IEC-Bmal1-/-
Naive: Bmal1flox
Naive: IEC-Bmal1-/-
DSS treated: Bmal1flox
DSS treated: IEC-Bmal1-/-

Figure S5: IEC-specific *Bmal1* deletion does not affect severity of acute DSS colitis. (A) Representative images of Periodic Acid Schiff (PAS) stained colon. **(B)** Number of goblet cells per crypt.

Figure S6

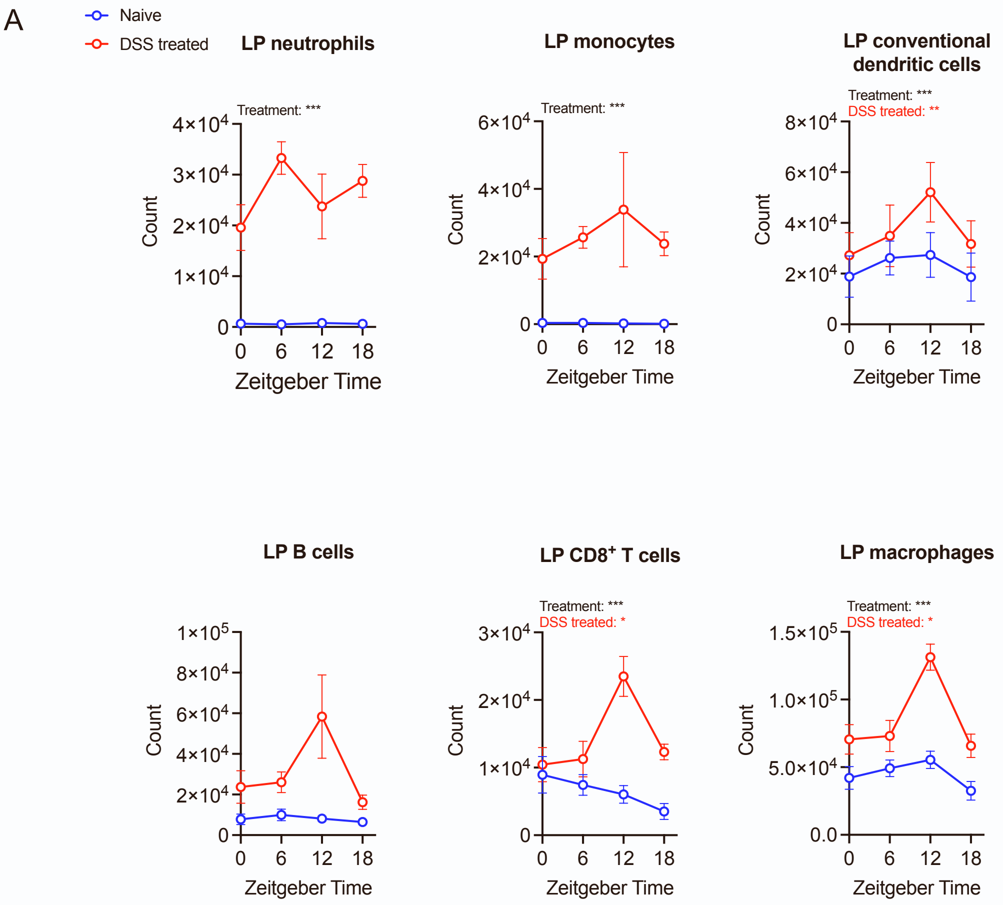

A
Naive
DSS treated
LP neutrophils
Treatment: ***
Count
Zeitgeber Time
LP monocytes
Treatment: ***
LP conventional dendritic cells
Treatment: ***
DSS treated: **
LP B cells
LP CD8+ T cells
Treatment: ***
DSS treated: *
LP macrophages
Treatment: ***
DSS treated: *

Figure S6: DSS treatment impacts lamina propria immune subsets. (A) Number of colonic lamina propria (LP) neutrophils (live $CD45^+Ly6G^+$), LP monocytes (live $CD45^+Ly6G^-CD11b^+CD64^+MHCII^-Ly6C^+$), LP conventional dendritic cells (live $CD45^+Ly6G^-CD64^-MHCII^+CD11c^+CD103^+CD11b^-$), LP B cells (live $CD45^+$ $CD19^+$), LP $CD8^+$ T cells (live $CD45^+CD3^+CD8^+$) and LP macrophages (live $CD45^+Ly6G^-CD11b^+CD64^+MHCII^+Ly6C^-$) determined by flow cytometry in wildtype mice (n=5/timepoint/treatment/genotype).

Statistics: (A) Two-way ANOVA with multiple comparisons (Šídák) and nonlinear regression to compare whether best fit is given by horizontal line or sine wave with nonzero baseline, constraints: wavelength = 24 hours; amplitude > 0. P value for sine wave displayed in coloured text on plots, where significant.

Figure S7

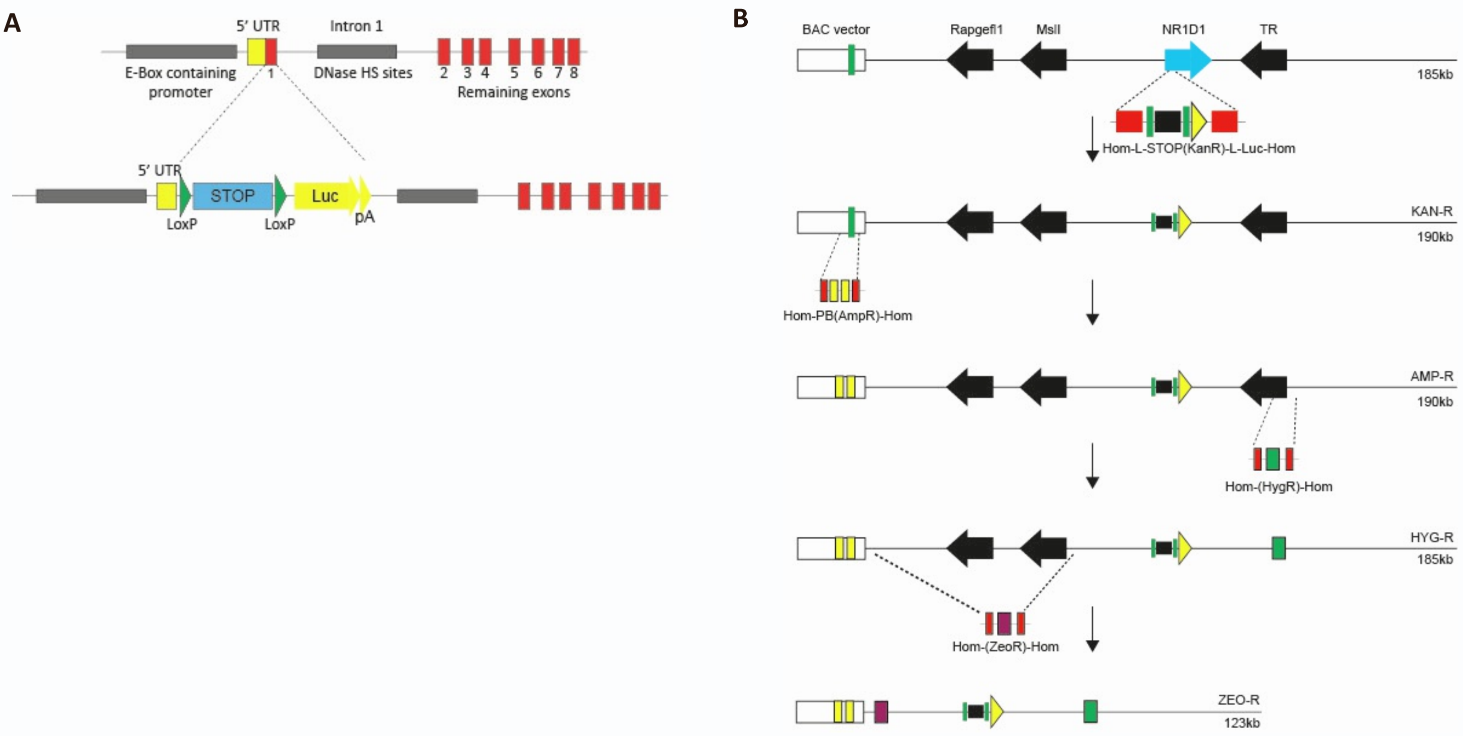
A
5′ UTR
Intron 1
E-Box containing promoter
1
DNase HS sites
2 3 4 5 6 7 8
Remaining exons
5′ UTR
STOP
Luc
LoxP
LoxP
pA
B
BAC vector
Rapgefl1
MslI
NR1D1
TR
185kb
Hom-L-STOP(KanR)-L-Luc-Hom
KAN-R
190kb
Hom-PB(AmpR)-Hom
AMP-R
190kb
Hom-(HygR)-Hom
HYG-R
185kb
Hom-(ZeoR)-Hom
ZEO-R
123kb

Figure S7: Generation of Nr1d1:Stop$^{fl/fl}$Luc mice. (A) Schematic of recombineering cassette to replace NR1D1 exon 1 with a conditional STOP site followed by Luciferase reporter gene. Note the presentation of key gene regulatory features including the upstream promoter, 5’UTR and intron 1 containing putative regulatory elements. **(B)** Overall view of BAC engineering including the integration of the reporter cassette, the PiggyBac ITRs in the BAC vector, and sequential removal of bystander genes.

Figure S8

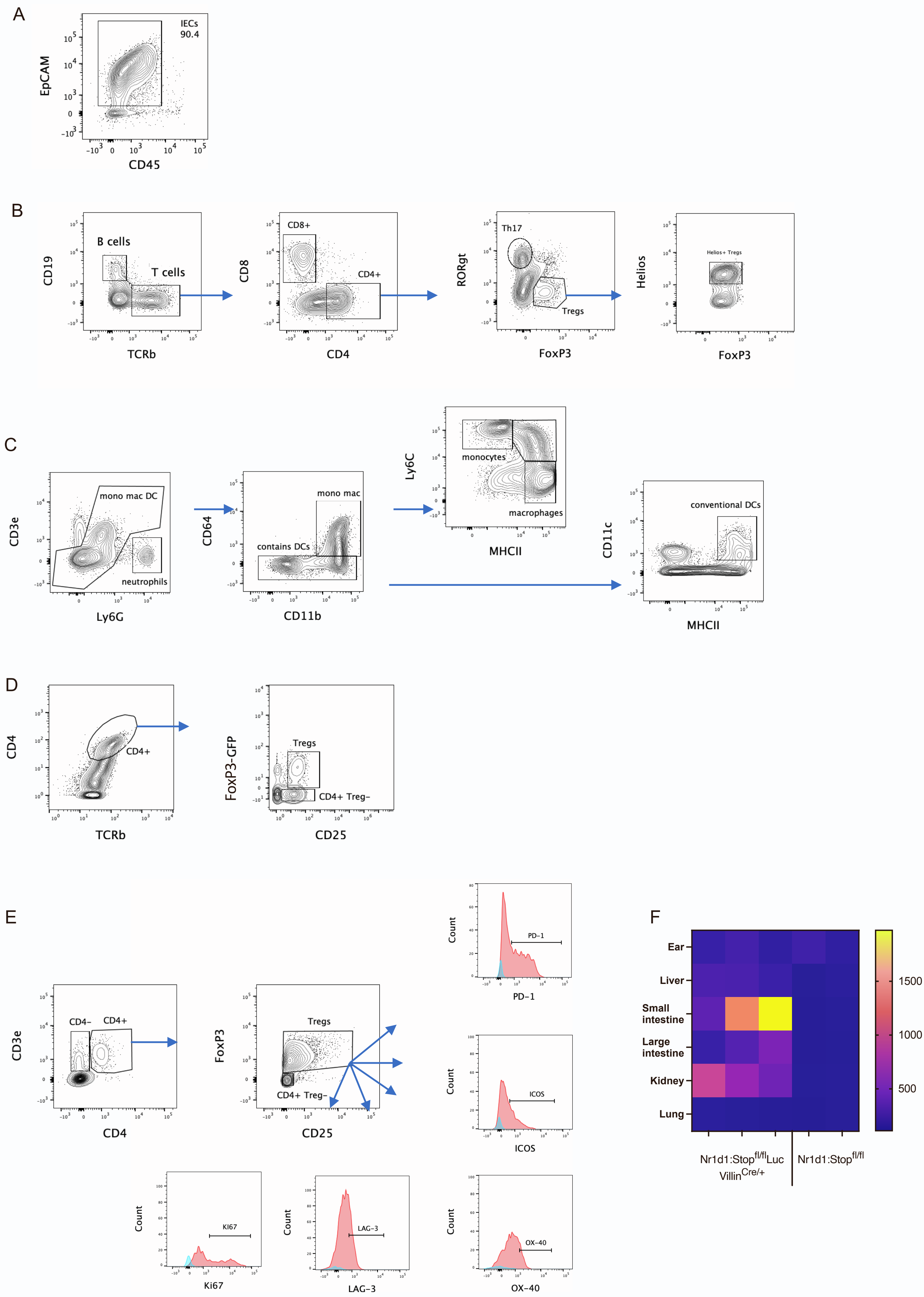

A
IECs
90.4
EpCAM
CD45
B
B cells
T cells
CD19
TCRb
CD8+
CD4+
CD8
CD4
Th17
Tregs
RORgt
FoxP3
Helios+ Tregs
Helios
FoxP3
C
mono mac DC
neutrophils
CD3e
Ly6G
mono mac
contains DCs
CD64
CD11b
monocytes
macrophages
Ly6C
MHCII
conventional DCs
CD11c
MHCII
D
CD4+
CD4
TCRb
Tregs
CD4+ Treg-
FoxP3-GFP
CD25
E
CD4-
CD4+
CD3e
CD4
Tregs
CD4+ Treg-
FoxP3
CD25
PD-1
Count
ICOS
Ki67
LAG-3
OX-40
F
Ear
Liver
Small intestine
Large intestine
Kidney
Lung
1500
1000
500
Nr1d1:Stop fl/fl Luc Villin Cre/+
Nr1d1:Stop fl/fl

Figure S8: Gating strategies for flow cytometry. (A) Flow cytometry data showing purity of the colonic intestinal epithelial cell (IEC) population ($CD45^{-}EpCam^{+}$) as purified by EDTA/DTT incubation. **(B)** Lymphoid gating strategy. Cells pre-gated as single/live/$CD45^{+}$. **(C)** Myeloid gating strategy. Cells pre-gated as single/live/$CD45^{+}$. **(D)** Gating strategy for regulatory T cells ($CD25^{+}GFP^{+}$) sorted from DEREG mice using the BD Influx. Cells pre-gated as single/live **(E)** Gating strategy for regulatory T cell functional panel. Cells pre-gated as single/live/$CD45^{+}$. Blue histogram is fluorescence minus one (FMO), pink histogram is representative sample. **(F)** Heatmap of total bioluminescent flux.
